# A Novel Nonsense Mutation of *ABCA8* in a Han-Chinese Family With ASCVD Leads to the Reduction of HDL-c Levels

**DOI:** 10.3389/fgene.2020.00755

**Published:** 2020-07-15

**Authors:** Chen-Yu Wang, Ya-Qin Chen, Jie-Yuan Jin, Ran Du, Liang-Liang Fan, Rong Xiang

**Affiliations:** ^1^Department of Cardiovascular Medicine, The Second Xiangya Hospital of Central South University, Changsha, China; ^2^Department of Cell Biology, School of Life Sciences, Central South University, Changsha, China; ^3^Hunan Key Laboratory of Animal for Human Disease, School of Life Sciences, Central South University, Changsha, China

**Keywords:** atherosclerosis, reduced HDL-c levels, cholesterol efflux, *ABCA8*, nonsense mutation

## Abstract

Arteriosclerotic cardiovascular disease (ASCVD) is one of the major causes of death worldwide and most commonly develops as a result of atherosclerosis (AS). As we all know, dyslipidemia is a leading pathogenic risk factor for ASCVD, which leads to cardiac ischemic injury and myocardial infarction. Dyslipidemias include hypercholesterolemia, hypertriglyceridemia, increased low-density lipoprotein cholesterol (LDL-c) and decreased high density lipoproteins cholesterol (HDL-c). Mutations of dyslipidemia related genes have been proved to be the crucial contributor to the development of AS and ASCVD. In this study, a Han-Chinese family with ASCVD was enrolled and the lipid testing discovered an obvious reduced levels of HDL-c in the affected members. We then performed whole exome sequencing to detect the candidate genes of the family. After data filtering, a novel heterozygous nonsense mutation (NM_007168: c.3460C>T; p.R1154X) of *ABCA8* was detected and validated to be co-separated in the family members by Sanger sequencing. Previous studies have proved that deleterious heterozygous *ABCA8* variants may disrupt cholesterol efflux and reduce HDL-c levels in humans and mice. This study may be the second report related to *ABCA8* mutations in patients with reduced levels of HDL-c. Our study not only contributed to the genetic counseling and prenatal genetic diagnosis of patients with ASCVD caused by reduced HDL-c levels, but also provided a new sight among ABCA8, cholesterol efflux and HDL-c levels.

## Introduction

Atherosclerosis is the major contributor of ischemic syndromes such as myocardial infarction or stroke, mainly resulted from plaque rupture and subsequent arterial blockade (Kobiyama and Ley, 2018). The incidence of AS is steadily rising along with an increasingly older population worldwide (Libby et al., 2016). The epidemiological survey shows that more than 20 million patients die from AS-related disorders worldwide every year (Herrington et al., 2016).

Previous studies have revealed that dyslipidemia was the predominant pathogenic factor of atherosclerotic plaque on the artery walls, which finally lead to AS and AS-related disorders (Hurtubise et al., 2016). And the main forms of dyslipidemia contains hypercholesterolemia, hypertriglyceridemia, and reduced high density lipoprotein (HDL-c). Dyslipidemia is a multi-factorial disease, which derives from complex interactions between genetic and environmental lesions. Many genes have been identified may be responsible for hypercholesterolemia and hypertriglyceridemia, for instance, *Low-Density Lipoprotein Receptor*, *Proprotein Convertase Subtilisin/Kexin type 9*, *Lipoprotein lipase*, *Apolipoprotein C2*, *Reticulon 3* and et al. (Surendran et al., 2012; Xiang et al., 2017, 2018). Low levels of HDL-c increase the risk of atherosclerotic cardiovascular disorder and shorten life expectancy. However, the underlying cause of reduced HDL-c values was still not clear. Several genes including ATP binding cassette subfamily A member 1 (ABCA1) and ATP binding cassette subfamily A member 8 (*ABCA8*) have been identified in patients with reduced HDL-c (Trigueros-Motos et al., 2017; Maranghi et al., 2019).

**FIGURE 1:**
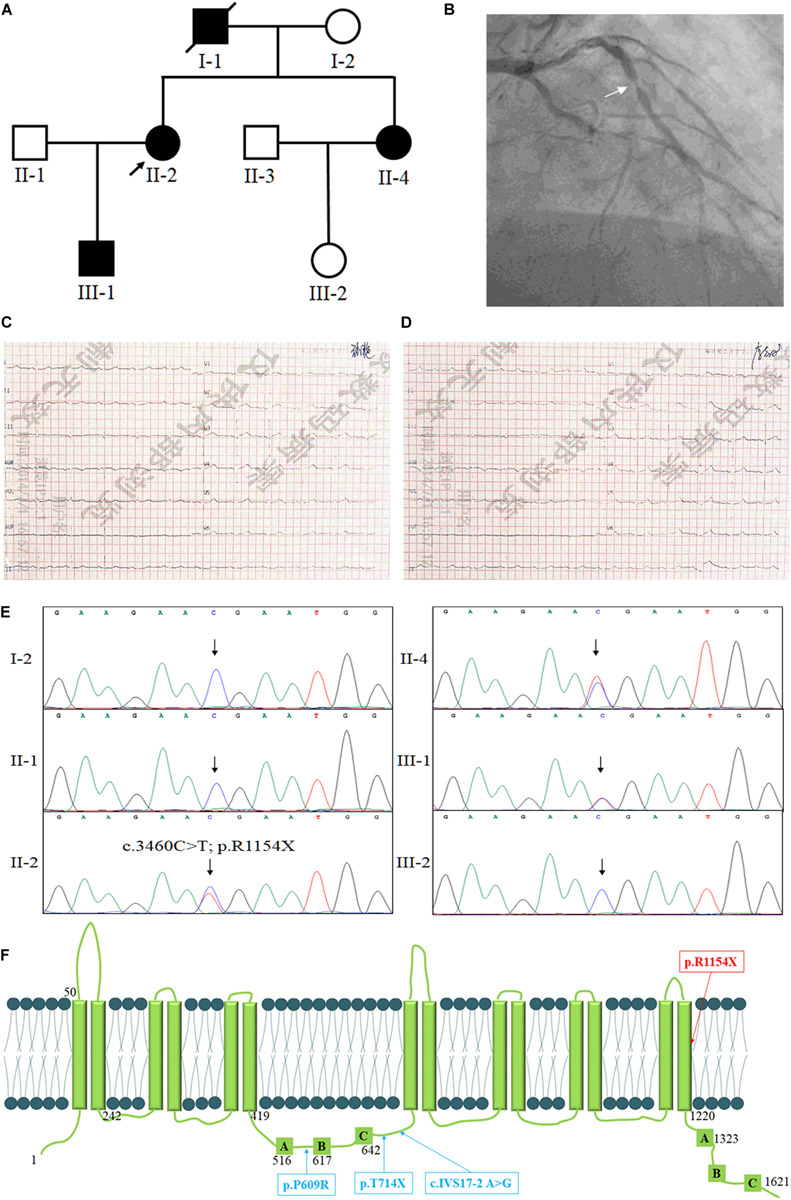
The clinical and genetic information of the family. **(A)** Pedigree of the family with low levels of HDL-c. Family members are identified by generations and numbers. Squares indicate male family members; circles, female members; closed symbols, affected members; open symbols, unaffected members; arrow, proband. **(B)** The coronary angiography of the proband, the arrow shows the stenosis of the anterior descending coronary artery. The ECG testing of the proband before percutaneous coronary intervention **(C)** and after percutaneous coronary intervention **(D)**. **(E)** Sequencing results of the *ABCA8* mutation. Sequence chromatogram indicates a C to T transition of nucleotide 3460. **(F)** The structure of ABCA8 and the summary of reported mutations of *ABCA8*.

Here, we enrolled a Han-Chinese family with Coronary Heart Disease (ASCVD) (Figure 1A). Lipid testing revealed that the levels of HDL-c were overt decreased, while the levels of low-density lipoprotein cholesterol (LDL-c), total cholesterol (TC) and triglycerides (TG) were close to normal standards. Whole exome sequencing and Sanger sequencing were employed to detect the genetic lesion of the family.

## Case Presentation

The proband (II-2), a 54-year-old lady, came to the hospital due to recurrent chest pain in last 2 years. Coronary angiography indicated approximately 60–80% stenosis of the anterior descending coronary artery (Figure 1B), ECG testing also suggested the patient suffered from CHD (Figure 1C). However, the lipid testing described the level of LDL-c (3.94 mmol/L; control: <3.12 mmol/L) and a distinctly reduced levels of HDL-c (0.41 mmol/L; control: 0.9–2.19 mmol/L) of the proband (Table 1). This discovery attracted our interest because most patients with ASCVD commonly presented high levels of LDL-c, TC, and TG (Hurtubise et al., 2016). We then investigated the family history of the proband (II-2), which indicated that her young sister (II-4) has been diagnosed as the occlusion of left iliac artery and her father (I-1) was died from myocardial infarction at 60-year-old. Lipid testing further described that both her son (III-1) and her young sister (II-4) showed an overt reduced levels of HDL-c and normal levels of LDL-c, TC, and TG (Table 1). And his young sister (II-4) also suffered from arterial plaque in the left lower limb. The blood pressure of the proband was 80–120 mmHg and the fasting blood-glucose was 5.2 mmol/L. The proband (II-2) accepted the treatment of percutaneous coronary intervention, the proband did not complain any uncomfortable after treatment. And breathing sound of the lungs was clearly, the heart rate was normal (Figure 1D), and the insertion site recovered well. The II-4 is accepting recovery treatment by exercise and diet control.

**TABLE 1:** The lipid testing data of the family members.

**Subjects**	**II-2 (proband)**	**I-2**	**II-1**	**II-3**	**II-4**	**III-1**	**III-2**	**Standard**
TC (mmol/L)	5.20	5.84	4.51	3.40	4.98	4.75	3.22	2.86–5.98
TG (mmol/L)	1.07	1.28	1.14	1.09	1.05	1.30	1.17	0.22–1.21
LDL-c (mmol/L)	3.94	4.32	3.96	2.77	4.02	3.99	3.05	<3.12
HDL-c (mmol/L)	0.41	1.19	1.43	1.38	0.47	0.51	1.60	0.9–2.19
AS and/or CHD	Yes	No	No	No	Yes	No	No	N/A
BMI (kg/m^2^)	22.48	23.55	22.49	23.71	22.20	24.07	24.34	18.5–23.9

*TC, total cholesterol; TG, triglycerides; LDL-c, low-density lipoprotein cholesterol; HDL-c, high density lipoprotein; AS, atherosclerosis; CHD, oronary heart disease; BMI, body mass index.*

## Laboratory Investigations

We supposed that the low levels of HDL-c may be the leading cause of AS and ASCVD in this family. However, what’s the genetic lesion underling the reduced levels of HDL-c in this family? We then isolated the genomic DNA of the proband and other family members (I-2, II-1, II-2, II-4, III-1, and III-2). Whole exome sequencing of the proband (II-2) was performed to detect the candidate gene of reduced levels of HDL-c.

In short, Exome capture and next-generation sequencing were conducted by Novogene Bioinformatics Institute (Beijing, China). One microgram of qualified genomic DNA from each person was captured by the Agilent’s SureSelect Human All Exon kit V5 (Agilent Technologies, Inc., Santa Clara, CA, United States) and sequenced by Illumina Hiseq4000 (Illumina Inc., San Diego, CA, United States). Shortly, genomic DNA were randomly carved by Covaris S220 sonicator (Covaris, Inc., Woburn, MA, United States) (Fan et al., 2019a). Then the fragmented DNAs underwent three enzymatic steps: end repair, A-tailing and adapters ligation. The adapter-ligated DNA fragments were amplified with Herculase II fusion DNA polymerase (Agilent). Later, the exomes in the pre-capture libraries were captured by SureSelect capture library kit (Agilent) (Fan et al., 2019b). After DNA quality estimation, the captured DNA library was used for next-generation sequencing on Illumina Hiseq4000 platform (Fan et al., 2019a). Downstream processing was carried out by Genome Analysis Toolkit (GATK), Varscan2 and Picard, and variant calls were performed by the GATK Haplotype Caller (Fan et al., 2019a). Variant annotation referred to Ensemble release 82, and filtering was conducted by ANNOVAR Documentation.

The filtered non-synonymous SNPs or INDELs with an alternative allele frequency more than 1% in public databases were kicked before further analysis. The public databases contains the NHLBI Exome Sequencing Project Exome Variant Server (ESP6500), dbSNP144^1^, the 1000 Genomes project^2^, the ExAC database^3^ and in-house exome databases of Novogene (2500 exomes) (Fan et al., 2019b). Then the filtered SNVs and INDELs, predicted by SIFT^4^, Polyphen2^5^, and MutationTaster^6^ to be damaging, were remained (Fan et al., 2019a).

After data filtering, a novel nonsense mutation (NM_007168: c.3460C>T; p.R1154X) of *ABCA8* was identified and validated by Sanger sequencing in the proband (Figure 1E). Previous studies have revealed that deleterious heterozygous *ABCA8* mutations may disrupt cholesterol efflux and reduce HDL-c levels in humans and mice (Trigueros-Motos et al., 2017; Sasaki et al., 2018). No other meaningful mutations related to lipid metabolism has been identified. Sanger sequencing further confirmed that only the affected individuals (II-2, II-4, and III-1) carried the novel nonsense mutation (NM_007168: c.3460C>T; p.R1154X) of *ABCA8* (Figure 1F). The novel mutation, resulting a truncated protein, was absent in the healthy members (I-2, II-1, and III-2) and 200 local people who were used as an internal control to exclude the SNP in local people (Fan et al., 2019a). Bioinformatics predicted that the newly identified mutation was deleterious and may disrupt the structure and function of ABCA8 (Schwarz et al., 2014). On the basis of ACMG guidelines (Richards et al., 2015), the novel variant meetings the following criteria from the ACMG guidelines: PVS1, PS3, and PM2.

## Discussion

As the extremely crucial transmembrane proteins, the ABC (ATP-binding cassette) transporters were encoded by 48 ABC transporter genes which were divided in to seven subfamilies named A–G in human (Kim et al., 2013; Hedditch et al., 2014). The ABC transporters are responsible for transferring substrates such as lipids, peptides, ions, carbohydrates, and vitamins across membranes by employing the energy from the hydrolysis of ATP (Trigueros-Motos et al., 2017; Sasaki et al., 2018). The subfamily A (ABCA) consists of 12 members in two subgroups: ABCA6-like and ABCA1-like transporter.

The subfamily A (ABCA) has 12 members with two subgroups, i.e., ABCA6-like and ABCA1-like transporter. The ABC1–4, 7, and 12 belongs to the ABCA1-like subgroup which play an important role in transporting cholesterol and phospholipids transport (Tsuruoka et al., 2002). However, less is known about the functional roles of the ABCA6-like subgroup transporters, i.e., ABCA5–6, 8–10 in humans, although several reports have described the tissue mRNA and protein expressions (Kim et al., 2013; Hedditch et al., 2014). ABCA8 is expressed in the brain, heart, small intestine, liver, lung, pancreas, prostate, spleen, testicle in human tissue (Bleasel et al., 2013; Demidenko et al., 2015; Gidding et al., 2015). However, less is known about its functional roles *in vivo*.

The human *ABCA8* gene encoding ATP-binding cassette-subfamily A, member 8 protein is located on chromosome 17q24.2, encoding 1621amino. ABCA8 contains 14 predicted transmembrane domains and 2 putative ATP-binding cassettes, but it lacks the common ABC transporter motif LSGGQ (Tsuruoka et al., 2002). At first, ABCA8 was classified into ABCA6-like transporters subgroup which was not responsible for lipid transporting in the ABCA family (Kaminski et al., 2001). However, ABCA8 was currently confirmed to associate with regulating cholesterol efflux and HDL-c levels in a similar fashion as the canonical cholesterol efflux proteins ABCA1 and adenosine triphosphate–binding cassette transporters G1 (Kim et al., 2008; Trigueros-Motos et al., 2017). Furthermore, ABCA8 has been suggested to associate with stimulating sphingomyelin production in oligodendrocytes (Bleasel et al., 2013; Kim et al., 2013). Meanwhile, the ABCA8 protein is reported to relate to ovarian cancer other than its role in anionic drugs transport across *Xenopus laevis* oocyte membranes (Hedditch et al., 2014; Liu et al., 2015). Here, we identified a novel nonsense mutation (NM_007168: c.3460C>T; p.R1154X) of *ABCA8* in a family with very low levels of HDL-c which further confirmed that deleterious heterozygous *ABCA8* mutations may disrupt cholesterol efflux and reduce HDL-c levels in humans. This discovery may be the second report related to *ABCA8* mutations in patients with reduced levels of HDL-c.

Commonly, increased LDL-c and TG were recognized as the crucial risk factors of ASCVD (Fernandez et al., 2015). Mutations of the pathogenic genes related to hypercholesterolemia and hypertriglyceridemia probably cause AS (Surendran et al., 2012; Xiang et al., 2017, 2018). Although reducing LDL-c levels has been proved to be an effective therapy, some patients still remain a high risk of ASCVD. This “residual risk” is majority result from elevated TG and low HDL-c levels (Fruchart et al., 2008). Our study, together with other researches, further revealed that the metabolism of HDL-c levels also played a significant role in ASCVD.

At present, only Trigueros-Motos et al. (2017) reported three deleterious heterozygous *ABCA8* mutations including p. P609R, c.IVS17-2 A>G and p. T741X in patients with reduced HDL-c levels (Figure 1F). In addition, the rs4148008 in *ABCA8* was reported to be significantly related to an average of 0.42 mg/dL HDL-c levels (Teslovich et al., 2010). In our study, the EXAC database contains three heterozygous carriers of the novel mutation, but we still believe that the novel mutation is the genetic factor of the family. Because the nonsense mutation is co-separated in the family members, bioinformatics analysis predict that this mutation is pathogenic and the mutation belongs to PVS1, PS3, and PM2 underling ACMG classification. It is reasonable when the novel mutation existed with an extremely lower MAF in EXAC database, since the reduced levels of HDL-c have a high risk of AS, but most symptoms of AS do not show up until a blockage occurs (Herrington et al., 2016; Kobiyama and Ley, 2018).

In mice, knockout *Abca8* may affect the efflux transporter for cholesterol and taurocholate. And the levels of HDL-c were significantly decreased in *Abca8* knockout mice. On the contrary, hepatic overexpression of human *ABCA8* in mice showed an obvious increased HDL-c in plasma (Trigueros-Motos et al., 2017). The phenotypes of human carried *ABCA8* mutations were consistent with the presentation in *Abca8* knockout mice. Here, the identified novel nonsense mutation (NM_007168: c.3460C>T; p.R1154X), resulting in the loss of function of ABCA8, also showed the same phenotypes with previous studies in human and *Abca8* knockout mice (Trigueros-Motos et al., 2017; Sasaki et al., 2018).

## Conclusion

In conclusion, we enrolled a Han-Chinese family with ASCVD. Lipid testing indicated overt reduced levels of HDL-c. Whole exome sequencing and Sanger sequencing detected a nonsense mutation (NM_007168: c.3460C>T; p.R1154X) of *ABCA8* in the ASCVD patients and absent in the healthy members. This study may be the second report related to *ABCA8* mutations in patients with reduced levels of HDL-c. Our study not only contributed to the genetic counseling and prenatal genetic diagnosis of patients with ASCVD caused by reduced HDL-c levels, but also confirmed the genetic lesion of the family with reduced HDL-c levels which suggested the family members with the novel mutation pay attention to ASCVD and accept medical examination regularly. In addition, our study also provided a new sight among ABCA8, cholesterol efflux and HDL-c levels.

## Data Availability Statement

The datasets for this article are not publicly available due to concerns regarding participant/patient anonymity. Requests to access the datasets should be directed to the corresponding author.

## Ethics Statement

The studies involving human participants were reviewed and approved by The Second Xiangya Hospital of the Central South University Ethics Committee. The patients/participants provided their written informed consent to participate in this study. Written informed consent was obtained from the individual(s) for the publication of any potentially identifiable images or data included in this manuscript.

## Author Contributions

C-YW and L-LF performed genetic analysis. Y-QC enrolled the samples and clinical data. J-YJ and RD isolated the gDNA and performed PCR. C-YW, Y-QC, and L-LF wrote the manuscript. RX supported the study. All authors reviewed the manuscript.

## Conflict of Interest

The authors declare that the research was conducted in the absence of any commercial or financial relationships that could be construed as a potential conflict of interest.

## Footnotes

1 http://www.ncbi.nlm.nih.gov/projects/SNP/index.html

2 http://www.1000genomes.org/

3 http://exac.broadinstitute.org

4 http://sift.jcvi.org/

5 http://genetics.bwh.harvard.edu/pph2/

6 http://www.mutationtaster.org/

## Abbreviations

ABCA1: ATP binding cassette subfamily A member 1
ABCA8: ATP binding cassette subfamily A member 8
AS: atherosclerosis
ASCVD: arteriosclerotic cardiovascular disease
HDL-c: high density lipoprotein
LDL-c: low-density lipoprotein cholesterol
TC: total cholesterol
TG: triglycerides.
